# Research trends and priorities in the treatment of psoriatic arthritis from 2014 to 2024: A bibliometric and visualization study

**DOI:** 10.1097/MD.0000000000048472

**Published:** 2026-04-24

**Authors:** Liwen Zhang, Fang Zhang, Diqian Zhao, Wenzhe Bai

**Affiliations:** a The First Clinical Medical School, Shandong University of Traditional Chinese Medicine, Jinan, China; b Department of Dermatology, Affiliated Hospital of Shandong University of Traditional Chinese Medicine, Jinan, China; c Department of Orthopedics, Affiliated Hospital of Shandong Traditional Chinese Medicine University, Jinan, China.

**Keywords:** bibliometric analysis, biologic therapies, psoriatic arthritis

## Abstract

**Background:**

Psoriatic arthritis (PsA) is a chronic immune-mediated inflammatory disease associated with psoriasis that can lead to progressive joint damage and impaired quality of life. Although biologic therapies targeting key inflammatory pathways have advanced rapidly, global research trends and hotspots in PsA treatment have not been systematically characterized.

**Methods:**

Publications on PsA treatment from 2014 to 2024 were retrieved from the Web of Science Core Collection. Bibliometric and visualization analyses were performed using CiteSpace and VOSviewer to evaluate publication characteristics, collaborative networks, and research trends, including keyword co-occurrence and citation burst analysis.

**Results:**

A total of 2704 publications were included. The United States ranked first in both publication output and citations, with extensive international collaboration, particularly with European countries, while contributions from the Asia-Pacific region have increased in recent years. Research focus has shifted over time from tumor necrosis factor inhibitors and clinical validation studies to interleukin-17/23 inhibitors, Janus kinase inhibitors, and personalized treatment strategies. Annals of the Rheumatic Diseases was the most influential journal, and Mease PJ was the most productive author. Keyword and citation analyses indicated that precision medicine, novel therapeutic targets, and combination therapies are emerging research directions.

**Conclusion:**

Research on PsA treatment has evolved toward precision and targeted therapy. Future studies should emphasize biomarker-guided treatment and optimization of combination strategies to improve clinical outcomes.

## 1. Introduction

In 1964, the American Rheumatism Association formally acknowledged psoriatic arthritis (PsA) as a distinct medical condition.^[1]^ PsA was originally defined by Moll and Wright as “ an inflammatory arthritis in the presence of psoriasis and usually in the absence of rheumatoid factor.”^[2]^ Prior to Wright seminal contributions, the prevailing view was that inflammatory arthritis occurring concomitantly with psoriasis was merely a coincidental manifestation of rheumatoid arthritis. PsA has been shown to result in a diminished quality of life, and a delayed diagnosis may lead to joint destruction and long-term disability. Therefore, it is imperative to recognize the key clinical features of PsA for the purpose of facilitating early diagnosis.^[3]^ PsA is characterized by the presence of joint pain, swelling and stiffness, often in an asymmetric distribution, and frequently involving the fingers, toes, and knees. The majority of patients also exhibit cutaneous manifestations of psoriasis, such as erythematous, scaly lesions. Nail lesions, including nail plate pitting, nail separation and subungual hyperkeratosis, are significant suggestive features of PsA.^[4]^ The treatment of PsA primarily relies on conventional drugs (e.g., disease-modifying anti-rheumatic drugs [DMARDs]) and novel therapies (e.g., biologics, small molecule drugs).^[5]^ There are 3 major classes of DMARDs, loosely grouped according to different mechanisms of action: conventional synthetic DMARDs such as methotrexate, sulfasala zine and leflunomide; biological agents (biological disease-modifying anti-rheumatic drugs) and targeted synthetic DMARDs, such as phosphodiesterase inhibitors or janus kinase (JAK)-inhibitors such as tofacitinib.^[6]^ As our understanding of the pathogenesis of PsA has improved, the use of biologics and small molecule drugs targeting specific inflammatory pathways has emerged as a means to improve patient outcomes. Biologics significantly reduce the inflammatory response and improve joint, skin and attachment point lesions by specifically targeting inflammation-related cytokines or receptors (e.g., tumor necrosis factor [TNF]-alpha, interleukin [IL]-12/23, IL-17, IL-23) and blocking disease-critical immune pathways. Biologics are more effective in reducing joint inflammation, minimizing psoriatic skin lesions, and preventing joint destruction than conventional drugs such as nonsteroidal anti-inflammatory drugs and DMARDs.^[7]^

Bibliometrics is a method of literature analysis that utilizes quantitative and qualitative analysis to examine the distribution structure, quantitative relationships, and evolving patterns of pertinent information within the literature. The objective of bibliometrics is to provide a comprehensive summary of the current status and developmental trends within a specific research field or medical condition. In addition, it aims to offer guidance to facilitate future progress in these domains.^[8]^ Bibliometric analysis, enhanced by modern computing, uses graphical and visual representations to strengthen literature reviews. Using CiteSpace and VOSviewer together leverages their strengths in producing knowledge graphs. CiteSpace applies set theory for data normalization, using specialized algorithms to create time-zone and timeline views, visualizing knowledge development over time. This approach highlights evolutionary patterns and emerging trends within a domain.^[9]^ VOSviewer uses a probabilistic approach to data normalization, offering visualizations for keywords, institutions, and coauthors. Its intuitive and visually appealing network, coverage, and density analyses are key features.^[10]^ Bibliometrics has been widely used in the field of psoriasis, such as psoriasis metabolomics,^[11]^ plaque psoriasis.^[12]^ Several prior bibliometric studies have examined psoriasis and PsA publications. While Berlinberg et al provided foundational insights into highly cited literature, their approach was citation-centric and lacked the temporal network analysis of biologic interventions.^[13]^ While Yu et al analyzed broad trends in psoriasis research, our study focuses specifically on biologic therapies in PsA and employs network-based clustering and temporal burst analysis to trace therapeutic evolution.^[14]^ In contrast, our study adopts a co-occurrence network approach with CiteSpace and VOSviewer, focusing on the evolution of therapeutic keyword clusters and citation bursts.

## 2. Materials and methods

### 2.1. Data collection

Web of Science has gained considerable traction among researchers as a digital literature resource database of exceptional quality, and it is widely regarded as the most suitable database for bibliometric analysis. In this study, Web of Science (Core Collection) was selected as the data source to ensure comprehensive and accurate retrieval of data by employing a strategy that incorporated both SCI-EXPANDED and SSCI indexes.^[15]^ We selected the Web of Science Core Collection (WoSCC) as the primary data source because it provides high-quality bibliographic information and citation data suitable for standardized bibliometric analysis. WoSCC is widely recognized for its curated indexing and is compatible with bibliometric tools such as CiteSpace and VOSviewer.

We conducted a literature search on January 1, 2025 in the WoSCC database related to PsA and biologic therapy. Our search strategy was as follows: TS=(“Psoriatic Arthritis” OR “PsA”) AND TS=(“biologic therapy” OR “biologics” OR “TNF inhibitors” OR “IL-17 inhibitors” OR “IL-23 inhibitors” OR “JAK inhibitors”).

**Figure 1.**
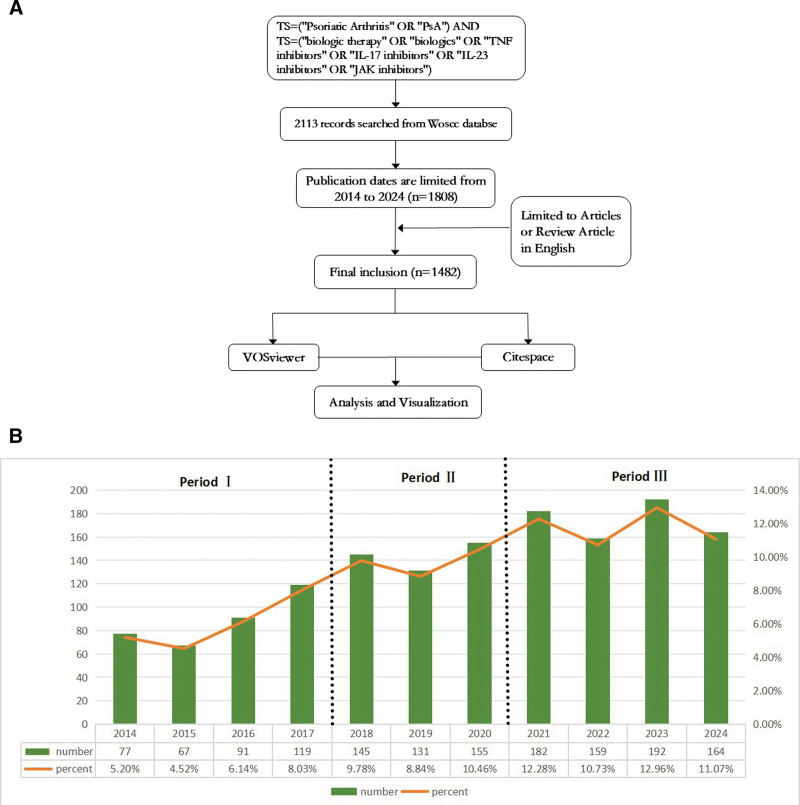
(A) Flowchart of the research. (B) Annual output of research on biologics for PsA treatment. PsA = psoriatic arthritis.

The language restriction is English, the time period is set to January 1, 2014 to December 31, 2024, and the document types are set to “Article” and “Comment.” A total of 1482 journal articles were obtained after automatic de-duplication using CiteSpace 6.3.R1. For details, see Figure 1A.

### 2.2. Data analysis

In this study, we used VOSviewer 1.6.10 and CiteSpace 6.3.R1 for bibliometric visualization and network analysis. For VOSviewer, the analysis included co-authorship, co-occurrence (keywords), citation, and bibliographic coupling networks. The minimum threshold for keyword co-occurrence was set to 5, and fractional counting was used. The layout algorithm was set to LinLog/modularity-based clustering, and the visualization type was network visualization. For CiteSpace, the time slicing was set from 2014 to 2024 with 1-year per slice. The term source included “title,” “abstract,” “author keywords,” and “keywords plus.” The node types selected were references and keywords. The selection criteria used the top 50 most-cited or most-frequent items per time slice. The pruning strategy included “Pathfinder,” “Pruning sliced networks,” and “Pruning the merged network.” The clustering algorithm used was log-likelihood ratio. These settings were selected based on standard practice and to enhance reproducibility.

VOSviewer 1.6.10.0 is a bibliometric analysis software that provides text mining functionality for constructing and visualizing co-occurrence networks of important terms extracted from scientific literature.^[16]^ In this study, the software performed the following analyses: country and institution analysis, journal and cited journal analysis, author and cited author analysis, and keyword co-occurrence analysis.

CiteSpace 6.3.R1 can be used to explore trends and dynamics of scientific research in specific research areas.^[17]^ In this study, CiteSpace was applied to map the dual-map overlay of journal and analyze reference with citation bursts to identify emerging themes.

## 3. Results

### 3.1. Overview of publications on PsA

A comprehensive search identified 1482 articles and reviews published between 2014 and 2024. The total number of citations was 35,427, with an average of 27 citations per document, and the collective H-index was 84. The entire period can be divided into 3 phases based on the annual growth rate of publications: a slow growth phase (2014–2017), a boom phase (2017–2020), and a stabilization phase (2020–2024).

As shown in Figure 1B, within Period I, the annual number of publications decreased from 77 in 2014 to 67 in 2015 and then gradually increased to 91 in 2017. This period is characterized by slow growth and significant fluctuations, with an overall lower trend. The number of papers published in Period II increased rapidly, from 91 in 2017 to 155 in 2020. The growth trend of publications in Period III has leveled off, and the annual number of publications has remained high.

### 3.2. Analysis of countries/regions

Table 1 presents the rankings of the top 10 nations and regions in biologic therapy for psoriatic arthritis. The United States (458 publications, 17,381 citations), Italy (280 publications), and the United Kingdom (232 publications) are the top 3 countries in terms of publication output. The U.S. also leads in H-index (84) and total citations.

**Table 1:** Top 10 productive countries/regions.

Rank	Country	NP	NC	H-index	Average citation peritem
1	USA	458	17,381	63	39.93
2	ITALY	280	7494	42	27.83
3	ENGLAND	185	10,083	50	55.64
4	GERMANY	136	5979	37	44.79
5	CANADA	124	5506	33	45.73
6	FRANCE	117	4753	34	41.55
7	SPAIN	107	5027	29	47.65
8	JAPAN	77	2264	24	30.27
9	SWITZERLAND	71	2231	27	32
10	NETHERLANDS	69	3446	23	50.36

**Figure 2.**
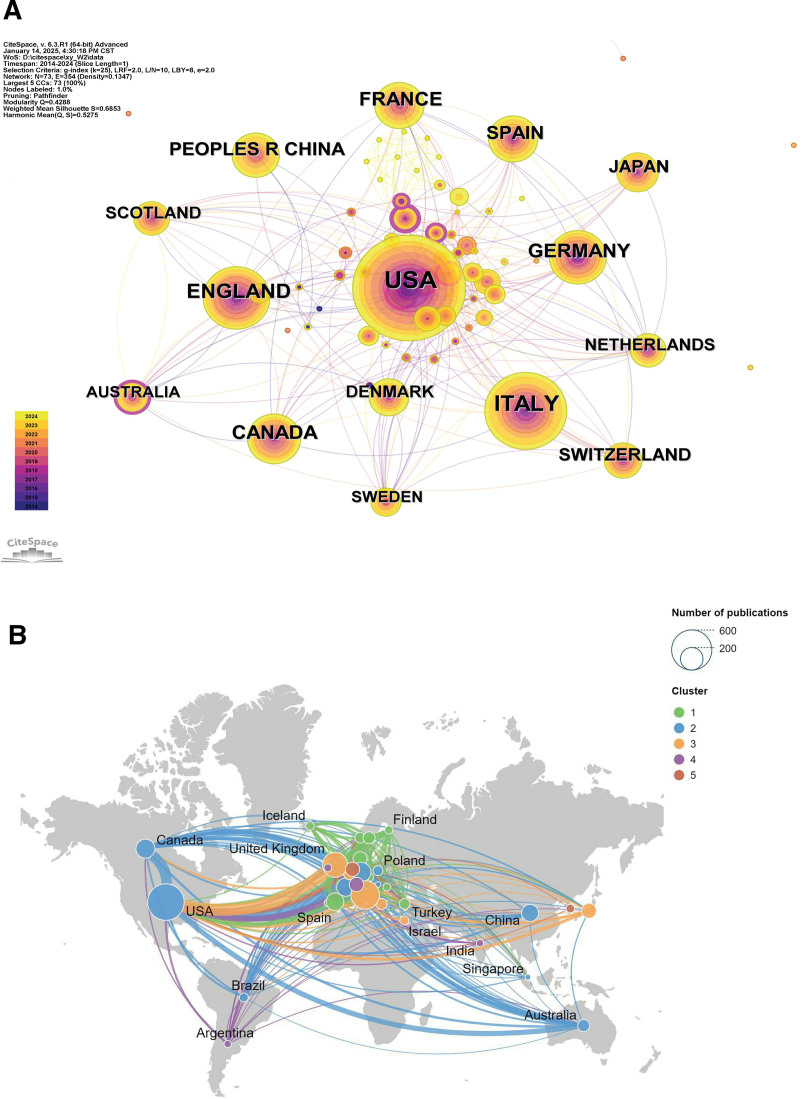
(A) Collaborative network analysis diagram for the treatment of PsA with biological agents. (B) Map of national collaborations on biologics for the treatment of PsA. PsA = psoriatic arthritis.

Figure 2A shows a map of country cooperation. In this map, nodes represent individual countries and different colors indicate different clusters. The size of the nodes reflects the number of articles published in that country. The connecting lines between the nodes indicate the cooperation between the countries, while the thickness of the lines represents the number of co-operations. As shown in the figure, the United States is the central hub in the network of co-operation in this field, and has established close co-operation with a number of countries around the world, including Canada, the United Kingdom, China, and Australia. A dense network of co-operation has been formed between European countries such as the UK, Germany, Italy, Spain, and Poland. The frequent cooperation between European countries indicates that Europe has a strong regional influence in this field. Attention should also be paid to the rise of the Asia-Pacific region, where countries such as China, Singapore, India, Australia, and others are occupying an increasingly important position in international cooperation. These countries are not only cooperating closely with the United States and Europe, but are also gradually forming a network of cooperation within the region. Countries such as Brazil, Argentina, Turkey, Israel, and Iceland are also actively involved in research in this field, which, although the scope of co-operation is relatively small, shows an expanding trend of global research in this field. Figure 2B shows an analyzed diagram of the international cooperation network, which also proves the above point. The United States, major European countries (e.g., Germany, Italy, and the United Kingdom), and Japan constitute the core international cooperation circle for PsA research.With the development of novel biologics research and immunotherapy, it is expected that international collaboration will further expand, especially with the participation of emerging research countries such as China and Australia.

Overall, research in this field is not only dominated by Europe and the United States, but is also gradually expanding to the Asia-Pacific region. Multinational collaboration has become an important trend in this field of research, helping to promote global advances in the treatment of psoriatic arthritis.

### 3.3. Analysis of affiliations

Table 2 shows the ranking of major institutions by publication volume. The University of California System ranked first with 74 publications, while the University of Washington recorded the highest H-index (28) and an average of 67.32 citations per article. Novartis (Switzerland) was the only company among the top institutions.

**Table 2:** Top 10 productive affiliations.

Rank	Affiliation	Country	NP	NC	H-index
1	UNIVERSITY OF CALIFORNIA SYSTEM	USA	74	2939	26
2	UNIVERSITY OF TORONTO	CANADA	59	2611	20
3	NOVARTIS	SWITZERLAND	57	1638	23
4, 5	UNIVERSITY OF WASHINGTON/SEATTLE	USA	57	3755	28
6	INSTITUT NATIONAL DE LA SANTE ET DE LA RECHERCHE MEDICALE INSERM	FRANCE	56	1746	22
7	UNIVERSITY OF COPENHAGEN	DENMARK	53	1330	21
8	SWEDISH MEDICAL CENTER	USA	52	3096	23
9	JEFFERSON UNIVERSITY	FRANCE	51	3014	23
10	ASSISTANCE PUBLIQUE HOPITAUX PARIS APHP	UK	51	3004	23

**Figure 3.**
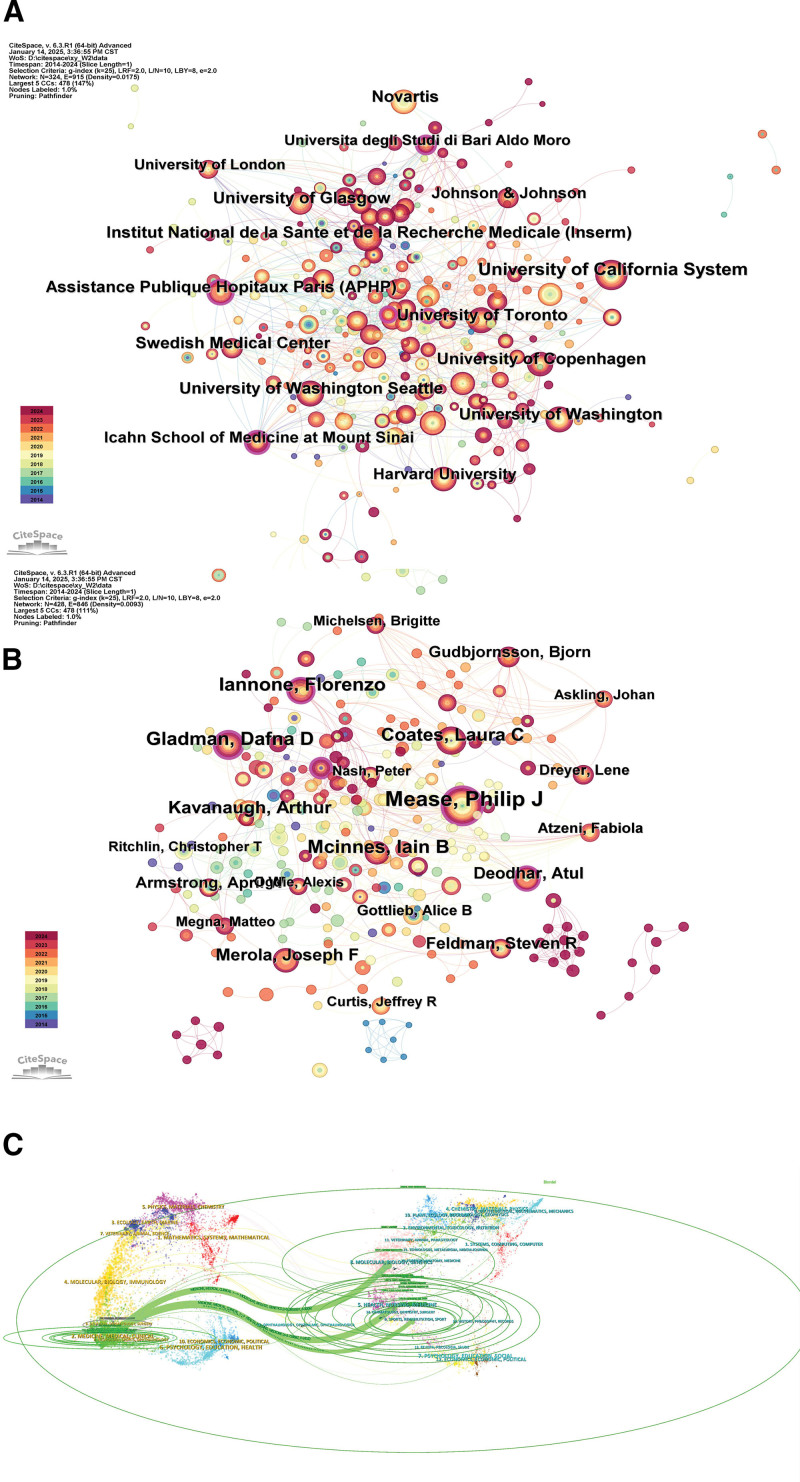
(A) Global network of research institutions collaborating on biologics for the treatment of PsA map. (B) Collaborative network of key scientists in biologics research map. (C) Double stacked diagrams of journals in biologics research. PsA = psoriatic arthritis.

As shown in Figure 3A, the University of California System, University of Washington Seattle, and Harvard University are the largest nodes of US institutions, demonstrating their significant contributions to the field of biologics for psoriatic arthritis. The University of California System and University of Washington Seattle are at the heart of the collaborative network with strong partnerships reflecting the dominance of the US in the field. Close collaborative networks have been formed between research institutions in Europe and North America, such as the University of Toronto (Canada), the University of Copenhagen (Denmark) and the Université de Paris (APHP) (France).The collaboration between the Institut National de la Santé et de la Recherche Médicale (Inserm) (France) and Johnson & Johnson (Pharmaceuticals) demonstrates the close links between academia and the pharmaceutical industry. This demonstrates the growing collaboration between pharmaceutical companies and academic research institutions to drive the development of therapeutic strategies. Institutions such as the Universityof California System, Harvard University, and the University of Toronto are increasingly prominent players in the development of new drugs and clinical trials.Research on novel biologics such as JAK inhibitors, IL-17, IL-23 inhibitors, etc will become a major future direction. The research of these new drugs will promote the development of precision therapy and individualized treatment strategies.

### 3.4. Performance of authors

Table 3 presents the top 10 authors, who collectively contributed 276 papers (approximately 18.62% of the total publications). Among these, 6 authors are from the United States. Mease, P.J. (Seattle University, Seattle) published 50 papers and achieved an H-index of 27. McInnes, Iain (University of Glasgow, England) and Iannone, Florenzo (University of Bari Aldo Moro, Italy) contributed 34 and 31 papers, respectively. Ritchlin, Christopher T. (University of Rochester Medical Center, Rochester) had an average of 104.36 citations per paper, and Gladman, Dafna (University of Toronto, Canada) recorded an average of 88.59 citations per paper.

**Table 3:** Top 10 authors with the most publications.

Rank	Author	Affiliation	Country	NP	NC	H-index	Average citation peritem
1	Mease, P. J.	Seattle University	USA	50	3567	0 27	72.74
2	McInnes, Iain	University of Glasgow	ENGLAND	34	2358	20	69.97
3	Iannone, Florenzo	University of Bari Aldo Moro	ITALY	31	589	13	19.39
4	Gladman, Dafna	University of Toronto	CANADA	27	2372	17	88.59
5	Kavanaugh, Arthur	University of California San Diego	USA	26	1539	14	59.73
6	Coates, Laura	University of Oxford	ENGLAND	24	1072	14	45.25
7	Deodhar, Atul	Oregon Health and Science University	USA	23	1783	17	78.39
8	Ritchlin, Christopher T.	University of Rochester Medical Center	USA	22	2276	18	104.36
9	Merola, Joseph F.	UT Southwestern Medical Center	USA	20	1111	13	56
10	Gudbjornsson, Bjorn	Landspitali University Hospital	ICELAND	19	251	8	14

As shown in Figure 3B, multiple nodes in the graph such as authors Philip J Mease, Dafna D Gladman, Iain B McInnes, and Arthur Kavanaugh form a core collaborative network of researchers who have had a significant impact in the field of psoriatic arthritis and biologics. Prof Philip J. Mease has many findings in the field of clinical applications of biological agents (e.g., TNF inhibitors, IL inhibitors) in the treatment of psoriatic arthritis.^[18]^ Prof Dafna D. Gladman is in the field of pathological research and clinical trials in psoriatic arthritis, with a special focus on the long-term effects and safety of biologics.^[19]^ Professor Iain B. McInnes’ research is centered on the study of immune mechanisms and the role of biologics (e.g., IL-17, IL-23 inhibitors) in psoriasis and psoriatic arthritis.^[20]^ He is a prominent figure in the field of cross-disease research. Prof Arthur Kavanaugh has made significant contributions to drug efficacy and drug retention studies in psoriatic arthritis, particularly in comparative studies of different biologics and the development of novel therapies.^[21]^

### 3.5. Analysis of journals

Table 4 details the distribution of publications across journals. *Annals of the Rheumatic Diseases* (IF = 20.3, 2867 citations) is the most influential journal. Other high-output journals include *Rheumatology*, *Journal of Rheumatology*, and *Clinical Rheumatology.*

**Table 4:** Top 10 most active journals.

Rank	Journal	IF (2023)	NP	NC	H-index	Average citation per item
1	RHEUMATOLOGY	4.7	70	2072	27	29.91
2	JOURNAL OF RHEUMATOLOGY	3.6	51	1189	21	23.57
3	CLINICAL AND EXPERIMENTAL RHEUMATOLOGY	3.4	43	575	16	13.67
4	CLINICAL RHEUMATOLOGY	2.9	41	520	14	12.88
5	JOURNAL OF DERMATOLOGY	2.9	34	423	13	13.32
6	RHEUMATOLOGY AND THERAPY	2.9	32	344	10	10.88
7	ANNALS OF THE RHEUMATIC DISEASES	20.3	30	2867	23	96
8	SEMINARS IN ARTHRITIS AND RHEUMATISM	4.6	30	787	17	26.4
9	EXPERT OPINION ON BIOLOGICAL THERAPY	3.6	29	364	13	12.69
10	EXPERT REVIEW OF CLINICAL IMMUNOLOGY	3.9	29	546	13	18.93

Figure 3C shows a double overlay diagram of journals, which is used to show citation relationships between journals in different subject areas and is a way to visualize the path of scholarly dissemination between journals. The green loops focus on clinical medicine (e.g., “Medicine, Medical Clinical”) and immunology (e.g., “Molecular Biology, Immunology”), showing the main disciplinary context of the study. The core journals are concentrated in the fields of Medicine, Immunology and Rheumatology, e.g. “Arthritis & Rheumatology,” “Journal of Rheumatology.” Biologics research is not limited to medicine, but also involves molecular biology, chemistry and other basic disciplines, indicating that the research is characterized by a high degree of multidisciplinary intersection.

### 3.6. Research hotspots, keywords analysis research hotspots analysis

**Figure 4.**
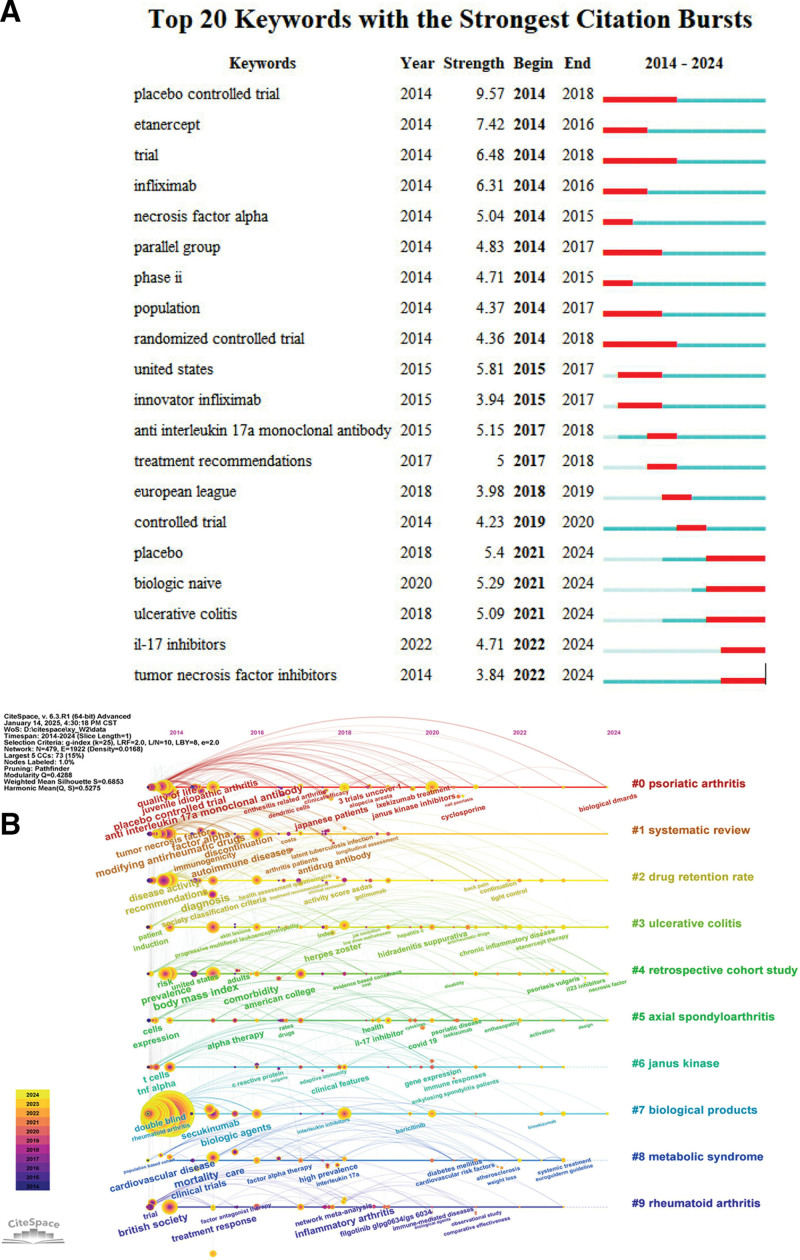
(A) Keywords highlighted in the field of biologics for the treatment of PsA. (B) Timeline diagram of the evolution of hotspots in the field of biologics for the treatment of PsA. PsA = psoriatic arthritis.

As shown in Figure 4A, the keyword emergence analysis can be divided into 3 phases.2014 to 2018: Studies were mainly focused on early biologics (e.g., Infliximab, Etanercept) and their clinical trials, with a focus on the efficacy of the drugs and controlled studies. 2015 to 2020: novel biologics such as IL-17 inhibitors begin to become a research hotspot, with a gradual shift in research toward drug efficacy, therapeutic recommendations, and the exploration of immune targets (e.g., IL-17). 2021 to 2024: research in biologic-naïve and co-morbid patients becomes the new hotspot, while IL-17 inhibitors and TNF inhibitors remain important regimens for the treatment of psoriatic arthritis.

To enhance thematic clarity, we grouped therapeutic-related keywords into 4 main biologic classes: TNF inhibitors (TNFi), IL-17 inhibitors, IL-23 inhibitors, and JAK inhibitors (JAKi). We then assessed their temporal distribution to illustrate the evolution of treatment focus in PsA research.Keywords associated with TNF inhibitors dominated earlier periods (2005–2015), reflecting their role as first-generation biologics.Since 2016, IL-17 inhibitors and IL-23 inhibitors have shown increasing co-occurrence, indicating growing research interest in novel IL-targeted therapies. Most recently, terms related to JAK inhibitors have emerged since 2019, suggesting the expansion of small molecule strategies.This temporal trend demonstrates a shift in therapeutic paradigms from broad TNF blockade to precision targeting of specific cytokine pathways.

Figure 4B shows the timeline of keywords in the field of biologics for psoriatic arthritis from 2014 to 2024. Each timeline corresponds to a keyword cluster, and the keywords are arranged along the timeline, showing the evolution and continuation of the research hotspots. The timeline mapping clearly demonstrates the evolution of the field of biologics for the treatment of psoriatic arthritis. The 2014 to 2016 study focused on efficacy and clinical trials with the goal of validating the efficacy of biologics in psoriatic arthritis. Research in 2016 to 2020 emphasizes comparative effectiveness and safety of biologics. JAK inhibitors are coming into view. In 2020 to 2024, the research focus will be on individualized treatment, long-term efficacy and safety (e.g., vaccination, metabolic risk). Novel therapeutic targets such as JAK and IL-17 inhibitors are gaining more attention. The overlay visualization clearly demonstrates temporal shifts in research focus. Emerging terms including “IL-23,” “JAK inhibitors”are shown in warmer colors, reflecting a surge in attention after 2020. These trends align with the clinical introduction and guideline endorsement of newer therapeutic agents and suggest a paradigm shift toward next-generation targeted therapies.

Notably, the emergence of keywords related to JAK inhibitors (e.g., “tofacitinib,” “upadacitinib,” “filgotinib”) coincides with key milestones in regulatory approvals and clinical integration. For example, tofacitinib was approved for PsA treatment by the Food and Drug Administration in 2017 and later by the European Medicines Agency in 2018. Upadacitinib and filgotinib received approval for PsA in subsequent years.This temporal concordance suggests that bibliometric trends in JAKi-related research reflect not only academic interest but also the impact of drug availability, clinical efficacy data, and formal guideline endorsement (highlighting a direct link between scientific discourse and therapeutic evolution).

### 3.7. Co-cited reference analysis

**Figure 5.**
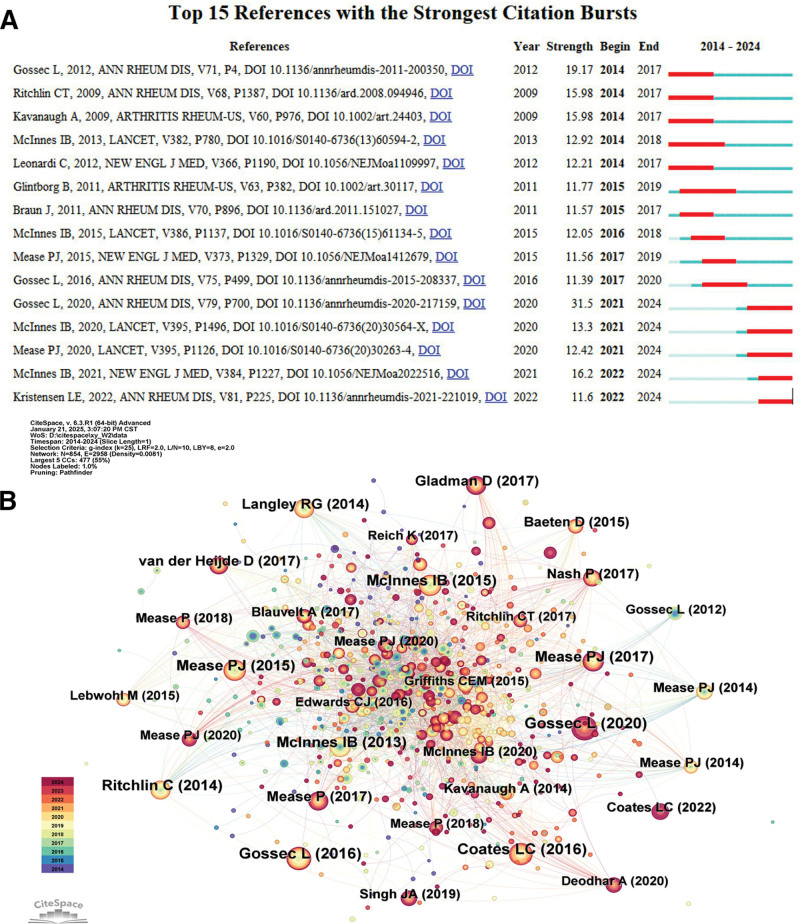
(A) Top 15 references with the strongest citation bursts of PsA. (B) Network map of author collaborations in the field of biologics for the treatment of PsA. PsA = psoriatic arthritis.

Figure 5A is a map of the prominence analysis of co-cited literature to show the trend of frequently cited literature and its research hotspots during 2014 to 2024. The citation burst detection function in CiteSpace identified several references with significant citation increases during specific time periods, reflecting the dynamic shifts in research focus and the emergence of new paradigms in PsA biologic therapy. For instance, the citation burst of Gossec et al^[20]^ between 2021 and 2024 (burst strength = 31.5) corresponds to the widespread adoption of updated European League Against Rheumatism treatment recommendations, signaling a paradigm shift toward incorporating novel biologics and treat-to-target strategies. Additionally, McInnes et al^[21]^ and Kristensen et al^[22]^ emerged as high-burst references in recent years, highlighting the growing attention to JAK inhibitors and IL-23 inhibitors, respectively. These represent the latest breakthroughs in immune-targeted therapies. In contrast, earlier high-burst papers such as Ritchlin et al^[21]^ and Kavanaugh et al^[23]^ reflect the initial enthusiasm around TNF-α inhibitors. This temporal citation burst pattern clearly delineates an evolution of research (from validation of early biologics to diversification into novel immune pathways and precision medicine). The burst analysis thus effectively captures the transformation of treatment strategies and emerging research frontiers. This evolution spans from the validation of the efficacy of TNF inhibitors,^[22]^ to the study of IL-17/IL-23 targets, and then to the exploration of JAK inhibitors. The research trend in this field reflects a gradual deepening and refinement process.

As shown in Figure 5B, McInnes IB appears several times in the atlas and is an important scholar in the field of psoriatic arthritis and biologics research, with his 2020 node in the literature being particularly notable. Mease PJ has published several highly cited papers covering the critical time period of 2014 to 2020. The center of the graph (e.g., literature by McInnes IB and Mease PJ) shows a dense network of co-citations, indicating that this literature is central to the field of biologics therapeutics. Peripheral nodes such as Langley RG (2014) represent older literature in basic research or subfields such as dermatology.

## 4. Discussion

### 4.1. General information

This study reveals the global research trends and hotspots of biologics for the treatment of psoriatic arthritis during the period 2014 to 2024 through bibliometric analysis. First, geographically, the United States and European countries dominate research in this field, especially the United States, which leads the world in terms of literature output, citation frequency, and academic impact (with the highest H-index and total citations). This indicates that the US and European countries play an important global leadership role in promoting the research and application of biologics for the treatment of PsA. Meanwhile, the Asia-Pacific region (e.g., China, India, and Australia) has seen a significant increase in research activity in recent years, demonstrating the region’s gradual rise in the global collaborative network. Second, the evolution of research hotspots shows a significant time-series feature. The field has evolved from the clinical efficacy validation of TNF inhibitors and IL-17 inhibitors in the early stage (2014–2018), to the exploration of immune targets (e.g., IL-23) in the middle stage (2018–2020), to the emerging hotspots in the near future (2020–2024), focusing on personalized therapy and long-term management. This shift in research hotspots reflects a gradual expansion from validating single-drug efficacy to managing overall patient health, aligning with the trend toward precision medicine.

### 4.2. Hotspots and frontiers

In terms of the use of biologics, previous studies have focused on the clinical efficacy and safety of TNF inhibitors. For example, Andreas Kerschbaumer et al screened a total of 2055 abstracts and ultimately analyzed 24 articles, including 15 observational studies and 9 long-term follow-up trials.^[21]^ With the discovery of novel targets such as IL-17 and IL-23, research has gradually expanded into the broader field of immunomodulation. Iain B McInnes et al’s study of Secukinumab, an IL-17A inhibitor, showed that Secukinumab subcutaneous injections demonstrated significant efficacy in improving the signs and symptoms of PsA patients, with a favorable safety profile.^[23]^ Both the keyword emergence analysis and literature co-citation analysis of this study further indicate that IL-17 and IL-23 inhibitors have become a research hotspot since 2018, validating the important role of related literature in advancing the field.

Our analysis shows that in recent years there has been a gradual shift in research towards personalized therapeutic strategies. This trend is consistent with the views of the UK expert panel on treatment strategies for PsA, highlighting the importance of individualizing treatment regimens according to patient characteristics, such as obesity or depression.^[24]^

In addition, there is a growing trend in research on drug combination treatment strategies. Previous studies have focused on the efficacy of single biologics, such as McInnes, Iain B et al’s study of Ustekinumab, which showed significant efficacy in improving PsA symptoms.^[25]^ And recent studies have begun to explore the potential value of combinations in improving the long-term prognosis of patients. For example, a study by Federico Diotallevi et al suggests that combining biologics with different mechanisms may provide a superior treatment option for some patients with refractory PsA. This study points to the need for more research to explore the long-term efficacy and safety of combination therapies, as well as the development of multi-targeted agents capable of treating multiple diseases simultaneously.^[26]^

### 4.3. Future directions and challenges

As medical research progresses, the limitations of a monotherapy regimen are gradually being exposed, especially with regard to differences in treatment response to biologics. It is expected that in the next decade, individualized treatment strategies will be further refined, particularly through the integration of biomarkers and genetic analysis to predict patient response to biologics. Biomarkers are biological molecules that reflect disease activity, response to treatment, or disease prognosis. For PsA, researchers have identified a number of possible biomarkers that can help predict a patient’s response to biologics.^[27]^ With the rapid development of genomics, genetic analyses offer the possibility of gaining insight into individual patient differences. Research suggests that the genetic background of PsA patients may play an important role in their response to biological agents.^[28]^ Personalized treatment for psoriasis will provide excellent results and minimize the risk of side effects.^[29]^ Through genomic analysis, doctors can predict which patients are most likely to benefit from a particular biologic and which patients may need to adjust their treatment regimen.^[30]^ Breakthroughs in genomics will drive the widespread use of precision medicine in the treatment of PsA, allowing for more personalized treatment and optimized outcomes.

Another important area of future research is the development of combination therapy strategies. PsA is a complex immune-mediated disease involving multiple pathways of the immune system, including cytokine networks, T-cell activation, and B-cell responses. In clinical care, while single therapeutic strategies (e.g., single biologics) are effective in many patients, for some patients, monotherapies often fail to completely control the disease, especially when dealing with complex inflammation and structural damage. Thus, combination therapy is emerging as a promising treatment option.^[31]^ Despite the clear advantages of combination therapy strategies, there are some challenges. For example, certain biological agents may increase the risk of infection when used in combination with immunosuppressive agents.^[32]^ Therefore, how to balance the therapeutic effect with side effects and how to choose the appropriate combination therapy regimen based on individual patient differences remain the focus of future research.

### 4.4. Limitations and prospects of the study

The bibliometric analyses for this study were conducted using the Web of Science Core Collection database. The analysis may not fully capture PsA-related publications indexed in other databases such as Scopus or PubMed, particularly those published in dermatology or immunology-specific journals. Although WoSCC is widely used in bibliometric research due to its standardized and high-quality citation data, future studies could incorporate multiple databases to improve the comprehensiveness and generalizability of the findings.

## 5. Conclusion

This paper provides a comprehensive overview of global research on biologic therapies for psoriatic arthritis over the past decade (2014–2024). The findings highlight the dominance of the United States and European countries in terms of publication output, citation impact, and collaborative networks, underscoring their leadership in advancing research on biologic therapies for psoriatic arthritis. Notably, there is a growing contribution from the Asia-Pacific region, reflecting the shift towards globalization of research. Key research hotspots have evolved from the early validation of TNF and IL-17 inhibitors to the more recent exploration of personalized therapies, new targets (e.g. IL-23, JAK inhibitors) and combination strategies. Incorporating biomarkers and genomics into treatment protocols is a promising frontier for precision medicine, while optimizing the safety and efficacy of combination therapies remains a challenge.

## 6. Software version and parameter settings

The software version and parameter settings used in the article are detailed in Table 5.

**Table 5:** Software version and parameter settings used in bibliometric analysis.

Software	Version	Analysis type	Key parameters
VOSviewer	1.6.10	Co-authorship, co-occurrence, citation	Min. keyword occurrences: 5; counting method: fractional; layout: LinLog/modularity clustering; visualization: network
CiteSpace	6.3.R1	Reference burst, keyword timeline, dual-map	Time slicing: 2014 to 2024; years per slice: 1; top N per slice: 50; pruning: Pathfinder, merged & sliced network pruning; clustering: LLR

LLR = log-likelihood ratio.

## Author contributions

**Conceptualization:** Liwen Zhang, Wenzhe Bai.

**Investigation:** Wenzhe Bai.

**Methodology:** Wenzhe Bai.

**Software:** Fang Zhang, Diqian Zhao.

**Supervision:** Fang Zhang.

**Validation:** Fang Zhang.

**Writing – original draft:** Liwen Zhang, Wenzhe Bai.

**Writing – review & editing:** Wenzhe Bai.
